# Open questions on the transition between nanoscale and bulk properties of metals

**DOI:** 10.1038/s42004-021-00466-6

**Published:** 2021-03-04

**Authors:** Rongchao Jin, Tatsuya Higaki

**Affiliations:** Department of Chemistry, Carnegie Mellon University, Pittsburgh, PA USA

**Keywords:** Electronic materials, Nanoparticles, Optical materials, Electronic properties and materials

## Abstract

Nanoscience has progressed tremendously in the exploration of new phenomena not seen in bulk materials, however, the transition between nanoscale and bulk properties is not yet fully understood. Here the authors identify and discuss remaining open questions that call for future efforts.

Metals (including alloys) are arguably the earliest man-made materials utilized for making tools after the Stone Age and have played critical roles in human civilization. Since the 20^th^ century, many mysteries of metals have been unraveled, such as their structures, mechanisms of electrical and thermal conduction, superconductivity, electronic excitations, quantum spin and exchange. Herein we focus on the evolution from atomic state to nanoscale to bulk metals, and the importance of fundamentally understanding where each regime begins and ends.

## Size evolution of metals from bulk to nanoscale

**Fig. 1:**
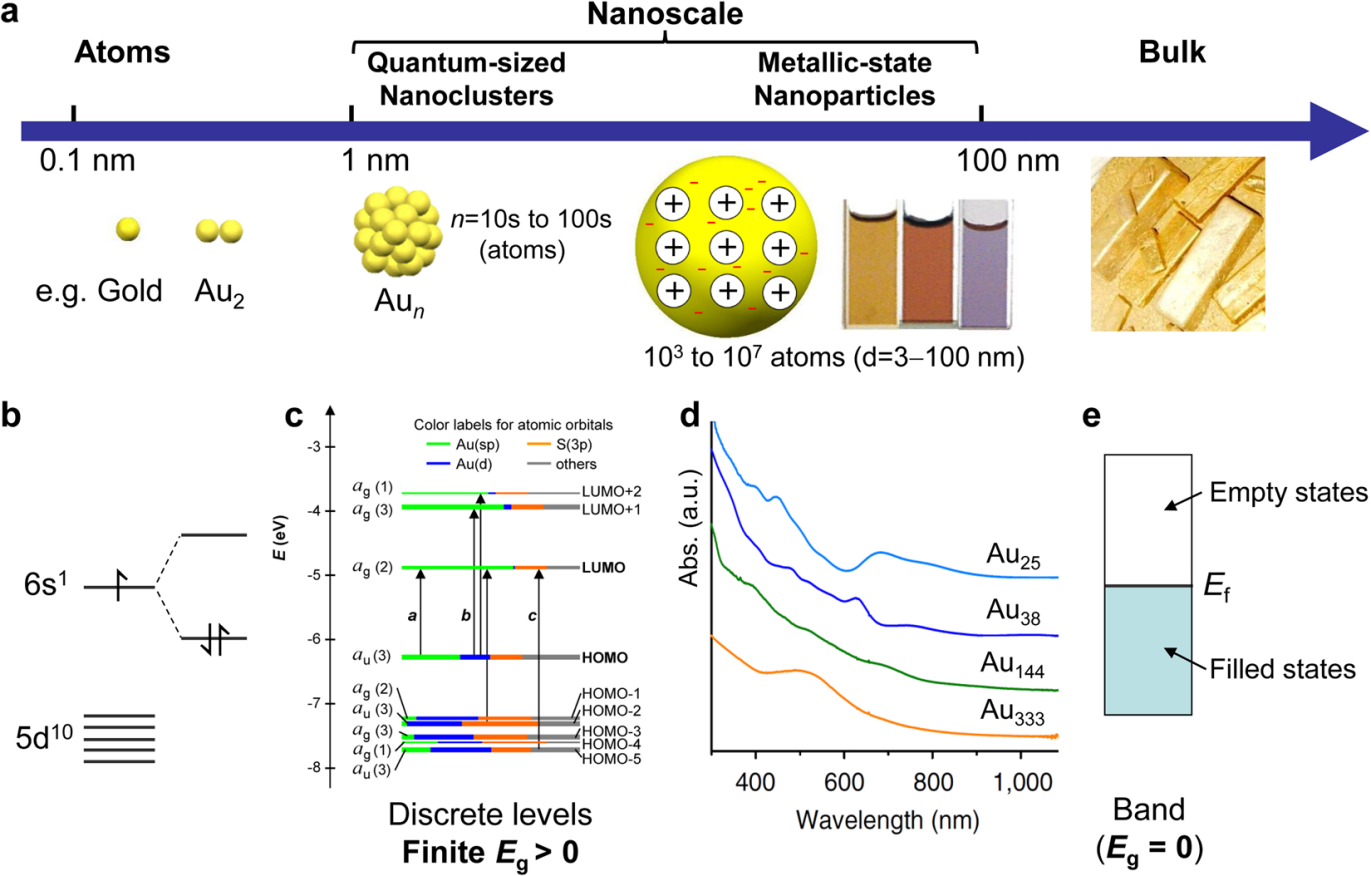
The evolution from atomic building blocks, to nanoscale, to bulk metals. **a** The nanoscale (1–100 nm) exhibits two distinct size regimes (quantum-sized: 1–3 nm (tens to hundreds of atoms), and regular metallic-state nanoparticles: 3–100 nm). **b** Atomic and diatomic electronic states. **c** Molecule-like electronic structure in quantum-sized nanoclusters (where, HOMO = highest occupied molecular orbital, LUMO = lowest unoccupied molecular orbital, *E*_g_ = HOMO-LUMO gap). **d** Evolution from discrete electronic excitation to collective electron excitation (plasmon) in optical absorption spectra with increasing size of nanoclusters. **e** Continuous band electronic structure of metallic-state nanoparticles and bulk metals (where, *E*_f_ = Fermi level/energy).

The size dependent evolution from bulk metals to the nanoscale is a central topic in nanoscience research (Fig. 1). Bulk metals are typically shinny, malleable and tractable, and they are highly conducting owing to the presence of freely roaming electrons. When the size of a metal is diminished to the micron scale (about the diameter of a human hair), not much change can be found in terms of the physical and chemical properties, albeit the surface-to-volume ratio is certainly increased. But further shrinking to the nanoscale (e.g., <100 nm) leads to significant changes to the properties; for example, surface plasmon resonances start to emerge in the nanoparticles (NPs) when interacting with light^1^. Over the size range of ~100 to ~3 nm, the optical properties of metal NPs are dominated by the surface plasmon resonances for both absorption and scattering of light, giving rise to beautiful colors of NPs^2^. Since the late 1990s, nanochemists have developed a variety of methods for achieving excellent control over NP size and shape, and demonstrated exquisite tailoring of the surface plasmon resonances of NPs by size and shape^2^.

Over the shrinkage from bulk to nanoscale (e.g., down to a few nm), while significant changes occur to the optical properties, the atom-packing mode and electronic-band structure in metals are essentially preserved^1–3^, even though the NPs are carved into different shapes with the assistance of ligands or stabilizers^2^. Using gold as an example, its crystal structure in bulk form is well known to be face-centered cubic (fcc), and this structure is retained over the size evolution from bulk to nanoscale before hitting the quantum size regime (see below)^1^, the same is true for the electronic-band structure, that is, metal NPs down to about 3-nm diameter still remain in a metallic state^1,3^. These aspects make the even smaller NPs (i.e., < 3 nm) quite intriguing, in particular the transition of atom-packing and electronic structures.

Another issue for NPs is that although high monodispersity has been achieved, they are not perfectly identical yet; for instance, no two NPs are the same at the atomic level^1^. This poses challenges to addressing some fundamental questions, such as the true surface structure of NPs, the core/ligand interface structure, and the exact surface composition; all these are critically important for understanding the electron transport, catalysis, and many other events^1^.

Motivated by the above fundamental issues, nanochemists further expanded their synthetic capabilities, and significant efforts over the past years have finally established the atomically precise nanochemistry, at least in the size regime of 1–3 nm, with hopes toward larger sizes in future efforts. The size-focusing synthetic methodology played a major role in creating a series of atomically precise NPs (often called nanoclusters for differentiating from conventional NPs)^1^. This methodology has been extended from gold to silver and alloy nanoclusters^1^.

At the bottom end of the nanoscale (Fig. 1, about 1–3 nm), quantum phenomena start to emerge, manifested in the transition from the electronic-band structure to discrete energy levels^4^ that are akin to those in molecules (Fig. 1). Such a quantization fundamentally alters the material properties and leads to emergence of many new phenomena^1^, such as the energy gap (*E*_g_), excitonic absorption of light, luminescence, unique catalytic activity, single-electron magnetism, and redox properties, to name a few. In addition to the electronic structure alteration, the atom packing structure also starts to exhibit significant changes. For instance, ultrasmall gold NPs are no longer exclusively fcc. Exotic structures of gold, such as body-centered cubic (bcc) and hexagonal close-packed (hcp) structures not present in bulk gold or larger NPs, have indeed been attained in ultrasmall sizes^1^. These exotic packing modes of gold atoms lead to extraordinary properties, such as the three-orders-of-magnitude variance in photoexcited electron lifetime^5^.

**Fig. 2:**
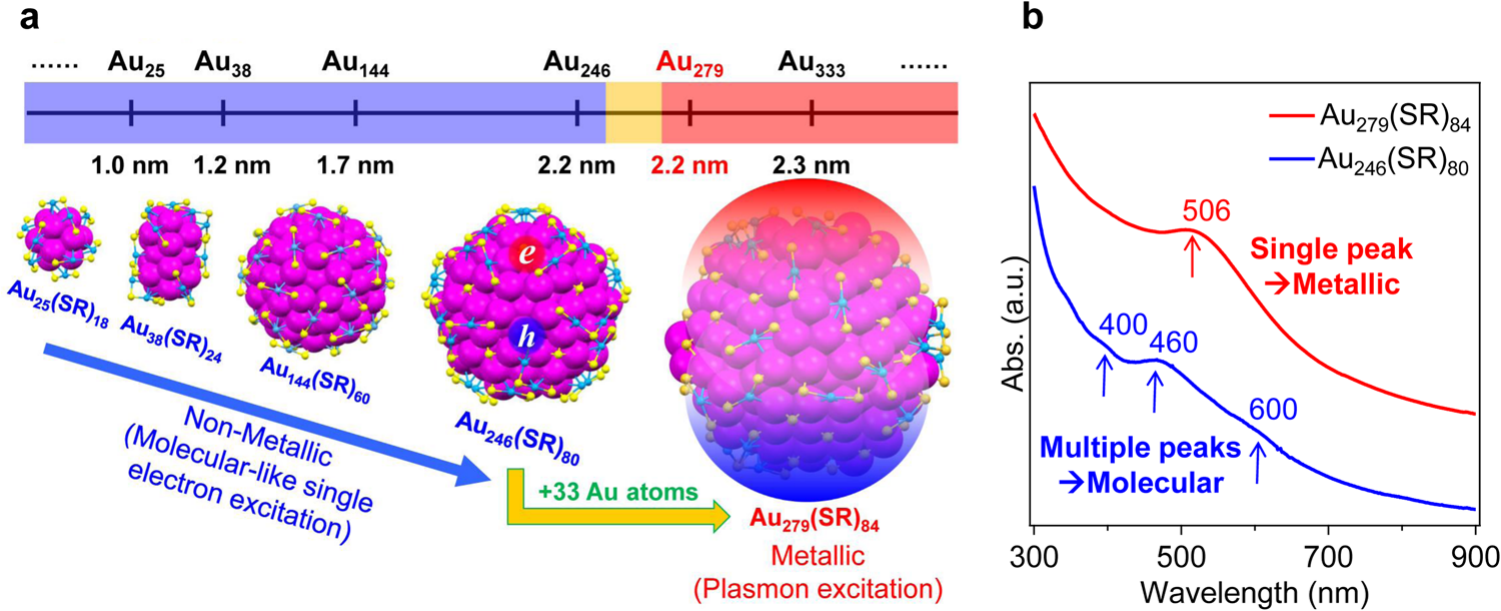
The size dependence of the electronic and optical properties in atomically precise gold nanoparticles. **a** Sharp transition from nonmetallic Au_246_ (bandgap *E*_g_ > 0) to metallic Au_279_ (*E*_g_ = 0). **b** Manifestation of the nonmetal-to-metal transition in optical absorption spectra.

The success of atomically precise nanochemistry has also led to insight into a decades-long fundamental question, that is, at what size the electronic band structure of metals (i.e. metallic state) evolves to discrete energy levels (i.e., semiconducting or molecular-like)^6–8^. This question came up in the 1930s shortly after the establishment of quantum mechanics, and it has since stimulated tremendous work in the physics and chemistry fields, but a major challenge was the synthesis of atomically precise NPs. With the advent of atomically precise nanochemistry, a sharp transition from metallic-state Au_279_(SR)_84_ (where, SR = thiolate ligands) to semiconducting Au_246_(SR)_80_ has recently been mapped out (Fig. 2)^8^, manifested in several aspects including the steady-state optical spectra^8^, femtosecond transient absorption^8^ and phonon dynamics^9^, as well as cryogenic spectroscopic features^8^. Back in the 1960s, Kubo^10^ raised a theoretical criterion for the metal-to-nonmetal transition, i.e., when *E*_g_ = *k*_B_*T* (where, *E*_g_ is the energy gap, *k*_B_ the Boltzmann constant, and *T* the temperature). This criterion indicates a temperature dependence and a smooth evolution, but neither was found experimentally^8^. Thus, the experimental results came as a surprise, which calls for a revisit to the electronic structure modeling of large-sized nanoclusters (of order ~10^2^ metal atoms). It should be pointed out that Kubo’s treatment did not include the electron–electron interactions^10^. Apparently, as the size grows, the density of electronic states increases (hence, smaller *E*_g_), and the electron correlation (e.g., screening) also becomes stronger, which ultimately leads to the collapse of discrete states and hence the emergence of the collective electron-gas.

## Open questions on the transition of electronic and optical properties of atomically precise metal NPs

Despite the discovery of the Au_246_-to-Au_279_ sharp transition, some open questions remain for future work. First of all, understanding the effect of shape on the transition is worth pursuing. Compared to the spherical cases of Au_246_ and Au_279_, nanoclusters of non-spherical shapes (e.g., one-dimensional rods, or two-dimensional oblate NPs) are more difficult to obtain; thus, new synthetic strategies are to be developed. The electronic transition in nonspherical cases could be more complex^11^ and remains to be investigated in future work. Second, it remains elusive to what extent the surface ligands affect the transition^1^. By changing thiolate ligands to other types, future work will reveal whether the surface ligands on nanoclusters possess any major effect on the transition. Third, to what extent does the detailed atom-packing structure (e.g., fcc, hcp, bcc and other types) influence the transition? The Au_246_ has a decahedral structure^7^, whereas Au_279_ is fcc^8^, thus, the potential effect of structure remains unclear. Last but not least, the phonon dynamics in transition-sized nanoclusters still remains elusive, which pertains to the electron-phonon coupling and the power dependence in probing the transition^6,9^. In particular, the scaling relationship of phonon frequency with the number of atoms in the nanocluster is not clear yet, and how this scaling evolves to the well-known phonon frequency~1/*d* law (where, *d* = diameter of NPs) in metallic/plasmonic NPs^3^. Future studies on the metal-to-nonmetal transition will promote fundamental understanding on the origin of metallic state and nascent plasmons^12^, and will also lead to the discovery of new properties of nanoclusters.

## Open questions on the catalytic properties of atomically precise metal NPs

Among the various applications of atomically precise metal NPs, catalysis constitutes a major topic^13^. Many open questions remain, such as the precise size effect at the atomic level, the true active-sites in catalytic processes, and the fundamental mechanistic steps^13^. Recent work has demonstrated the promise of atomically precise NPs in pursuing such aspects^14^. When comparing different nanocatalysts, often many factors come into play simultaneously, for example, the investigation on the size effect at the atomic-level involves different-sized NPs, but other than the size, the structures or the surface ligands would often become different as well, which complicates the analysis of the size effect. For future work, key efforts should focus on the creation of correlated nanocatalysts with only one factor changed while other factors are kept the same (e.g., a correlated pair of nanoclusters with the same core but different surfaces)^15^. Such correlational studies will be extremely important for studying the effects of size, structure, composition, ligand, interface, and other factors in a manner of one at a time, rather than multiple factors being entangled. In revealing the catalytic mechanisms, site-specific tailoring approaches hold great promise, for instance, a local surgery for replacing the surface motif^13^. Another approach is the single-atom-level tailoring in the core, that is, one atom at a time to observe the catalytic effect of heteroatom doping^14,15^. By combining experiment and theory, future work is expected to unravel the fundamental principles of synergy in atomically precise nanoalloys.

## Outlook

Finally, while we focus on metal NPs in the above discussions, semiconductor and magnetic NPs also have some remaining fundamental issues. Atomic precision should be pursued in future research in order to solve the issues of ambiguous surface composition and interface structure of quantum dots and magnetic NPs. With atomically precise NPs, there will also be new opportunities in assembling NPs into coherent artificial solids, which will open up new opportunities in research.

Overall, as a new class of nanomaterials, atomically precise NPs are expected to impact the fundamental research of nanoscience, in particular, the structure-property relationships at the atomic level. Based upon the new knowledge acquired, atomically precise nanomaterials will find new opportunities in a wide range of applications. Looking into the future of nanoscience, atomically precise nanochemistry will open up many exciting opportunities.
